# Natural Cyanobacteria Removers Obtained from Bio-Waste Date-Palm Leaf Stalks and Black Alder Cone-Like Flowers

**DOI:** 10.3390/ijerph19116639

**Published:** 2022-05-29

**Authors:** Irina Kandić, Milan Kragović, Jugoslav B. Krstić, Jelena Gulicovski, Jasmina Popović, Milena Rosić, Vesna Karadžić, Marija Stojmenović

**Affiliations:** 1 “Vinča” Institute of Nuclear Sciences, National Institute of the Republic of Serbia, University of Belgrade, 11351 Belgrade, Serbia; irina.kandic@vinca.rs (I.K.); m.kragovic@vinca.rs (M.K.); rocenj@vinca.rs (J.G.); mrosic@vinca.rs (M.R.); 2 Centre for Catalysis and Chemical Engineering, National Institute, Institute of Chemistry, Technology and Metallurgy, University of Belgrade, 11000 Belgrade, Serbia; jkrstic@nanosys.ihtm.bg.ac.rs; 3 Department of Chemical and Mechanical Wood Processing, Faculty of Forestry, University of Belgrade, Kneza Višeslava 1, 11030 Belgrade, Serbia; jasmina.popovic@sfb.bg.ac.rs; 4 Institute of Public Health of Serbia Dr. Milan Jovanović Batut, dr Subotića 5, 11000 Belgrade, Serbia; vesna_karadzic@batut.org.rs

**Keywords:** activated carbon, date palm, black alder, cyanobacteria, adsorption capacity

## Abstract

The impact of urbanization and modern agricultural practice has led to accelerated eutrophication of aquatic ecosystems, which has resulted in the massive development of cyanobacteria. Very often, in response to various environmental influences, cyanobacteria produce potentially carcinogenic cyanotoxins. Long-term human exposure to cyanotoxins, through drinking water as well as recreational water (i.e., rivers or lakes), can cause serious health consequences. In order to overcome this problem, this paper presents the synthesis of completely new activated carbons and their potential application in contaminated water treatment. The synthesis and characterization of new active carbon materials obtained from waste biomass, date-palm leaf stalks (P_AC) and black alder cone-like flowers (A_AC) of reliable physical and chemical characteristics were presented in this article. The commercial activated carbon (C_AC) was also examined for the purpose of comparisons with the obtained materials. The detailed characterization of materials was carried out by X-ray diffraction (XRD), Fourier-transform infrared spectroscopy (FTIR), low-temperature N_2_ physisorption, and Field emission scanning electron microscopy (FESEM). Preliminary analyzes of the adsorption capacities of all activated carbon materials were conducted on water samples from Aleksandrovac Lake (Southern part of Serbia), as a eutrophic lake, in order to remove Cyanobacteria from water. The results after 24 h showed removal efficiencies for P_AC, A_AC, and C_AC of 99.99%, 99.99% and 89.79%, respectively.

## 1. Introduction

The rapid growth of population, urbanization, and modern agricultural practices has led to the accelerated eutrophication of aquatic ecosystems. Shallow lakes and reservoirs are mostly exposed to this process, where the water retention time is extended, the accumulation of nutrients is increased, and the trophic status affects large biomasses of algae and Cyanobacteria. These all lead to the process known as “water blooms”. The process of eutrophication favors the proliferation of Cyanobacteria rather than the water blooms of eucaryotic algae [1]. Over the last few decades, the frequency of water blooms due to the large number of Cyanobacteria has become more widespread. It is especially necessary to emphasize the importance of the presence of certain species as potential producers of toxins, i.e., cyanotoxins. Therefore, the problem of widespread Cyanobacteria blooms has become an increasingly important and current topic because it affects public health. The effect of human exposure to cyanotoxins can be hepatotoxic, neurotoxic, or genotoxic, and long-term exposure to these cyanotoxins can be lethal [2]. It should be noted that it is difficult to predict environmental factors that affect the release of cyanotoxins into the water, which is the main reason for finding adequate treatment method(s) which will remove the potential producers of cyanotoxins. In the available literature, different methods for the removal of cyanobacteria cells from water are presented. Modern water treatments provide for the physical removal of cells as well as the removal of dissolved toxins. However, many of those water treatments are still not sufficiently developed. That can lead to water pollution and, therefore, can harm ecosystems and human health. The conventional water treatment processes are coagulation, flocculation, sedimentation, filtration, and sludge dewatering [3]. Specifically, until recently, activated carbon materials have been used for cyanotoxins’ removal; however, they were not popular for cyanobacteria removal, such as microcystin and cylindrospermopsin removal [4,5]. Activated carbon materials are good adsorbents due to their easy preparation and controlled pore structure, high thermal stability, good chemical resistance, low regeneration energy, low sensitivity to water, and low cost, and their hydrophobicity properties allow a very diverse application [6].

The choice of the precursor for the permanent synthesis of activated carbon materials depends on their availability in nature, yield of activation, and adsorption efficiency for pollutant removal. The development of modern society induced a fast-growing production of activated carbon materials and their application due to strict environmental regulations in the area of water resources. Therefore, it is important to find the type of precursor that is renewable and abundant in nature. In order to find renewable and low-cost alternatives for the production of activated carbon from by-products and biomass residues, the need for finding suitable material increased in recent years. Compared to traditional activated carbon, one of the main advantages of activated carbon materials derived from alternative precursors is their high availability and low cost [7]. The lignocellulose content of the precursor is an important parameter, because the cross-linking between the lignocellulose components determines the suitability of biomass material for activated carbon material [8]. The lignocellulose content includes a percentage of lignin, cellulose, and hemicellulose. It is noticed that the precursors with a high content of lignocellulose have been proven to be good and inexpensive precursors to use to produce activated carbons [9]. In addition to the mentioned composition of activated carbons, their structural characteristics for their application are also important, as well as their high porosity and surface area [10].

The precursors that were used in this paper for the preparation of activated carbon materials are date-palm leafleaf stalks (*Phoenix daclylifera* L.) and black alder cone-like flowers (*Alnus glutinosa* L.). In the available literature, there are papers where both precursors were characterized using different techniques [11,12,13]. However, there are no data on obtaining activated carbons from these precursors and their specific applications on the treatment of real contaminated waters. The research describing date palms is presented in papers Khiari et al., 2010 and Altobai et al., 2019 [11,12]. Its various parts are described in the papers which were examined, as well as the climate and area which can affect the characteristics of the plant [12]. On the other hand, the research about black alder is still developing and is described in paper Mokrzycki et al., 2020 [13]. In this paper, the differences between samples of carbonized cone-like flowers and wood chips at different temperatures from 200 to 800 °C, as well as lignocellulose content levels, are presented. However, until now there has not been any potential uses of leaf stalks for preparing activated carbon materials.

**Figure 1:**
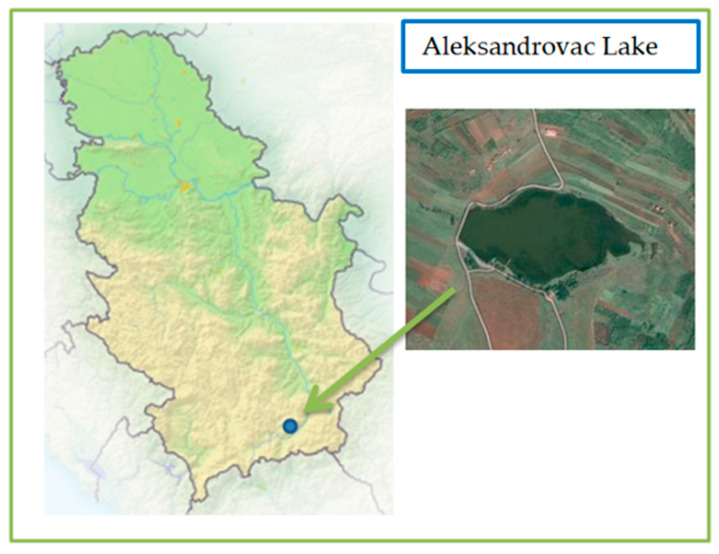
Satellite view of Aleksandrovac Lake, Serbia.

The main aim of this paper and the difference between it and other published papers is the application of different methods for the synthesis of activated carbon material by using date-palm leaf stalks and black alder cone-like flowers. In the paper, their physical–chemical characteristics, as well as their potential applications in order to remove Cyanobacteria from contaminated waters, were analyzed and compared. In particular, the detail physical-chemical characteristics of the obtained activated carbons will be present in this paper and their benefits for potential application for the determination of adsorption properties will be considered. In order to determine the adsorption properties of materials and their potential application for water purification, preliminary analysis was performed on a water sample contaminated with Cyanobacteria from high eutrophic lake from Southern part of Serbia, Aleksandrovac Lake (artificial reservoir; 42°29′22″ N, 21°53′54″ E; Figure 1).

Over time, due to increased activity and inflow of nutrients in the basin, there was an intensive eutrophication of the Aleksandrovac lake. Eutrophication and climate change are two of the most common factors that can affect the increase in volume, population density, and persistence of cyanobacteria blooms which could result in fish mortality [14]. There were two massive fishing mortalities in 2008, with a consequent loss of over 3000 kg of the fish. The possible reason was a change in the pH of lake water and a lowering oxygen concentration, as well as toxin production by cyanobacteria [15]. Rapid eutrophication leads to forced efforts for improving the water quality, and the reconstruction program began in the spring of the 2010 [16]. The lake was refilled with water and then used for tourism and recreation during the summer of 2010. However, a massive new fishing mortality in Aleksandrovac Lake was in mid-December 2012. Several weeks before the incident, a cyanobacterial species Cylindrospermopsis raciborskii was noted, which gave a yellow/brown color to the water. The tropical potentially invasive toxic cyanobacteria species Raphidiopsis raciborskii (Woloszynska) Aguilera et al., 2018 (formerly Cylindrospermopsis raciborskii (Woloszynska) Seenayya and Subba Raju 1972) is known for spreading areal into moderate climate regions. R. raciborskii produces several toxins: cilindrospermopsin, saxitoxins, and anatoxin [17]. The way these species can survive in moderate climates is by ackinetes (dormant cells of cyanobacteria species), which help it to tolerate unfavorable conditions and are the main cause of their invasion [18]. For the first time, this species was recorded in Serbia and was described in 2006 by Cvijan and Fužinato et al., 2011, in Slatina Pond [19]. Its first blooming was recorded in Ponjavica river in 2008 [20]. The control of the presence and abundance of this species in many countries is extremely important for water quality management, since the proliferation of this species never forms a surface bloom, so it cannot be detected by visual monitoring. The R. raciborskii shows wide tolerance to temperature changes; it is capable to sustain biomass at temperatures as low as 11–17 °C, and up to 35 °C, which was the highest temperature that was observed when the population increased [21]. Due to the presented results, this lake was chosen as an example of a lake suitable for testing the possibility of the application of new activated carbon materials.

Therefore, convenient and inexpensive methods of synthesis that enable the production of activated carbons with a precisely defined structure necessary for their specific application in purification of contaminated water are key to the development of these materials, which is the main goal of this paper.

## 2. Materials and Methods

### 2.1. Materials

In this study, two precursors in form of raw samples were used for the preparation of new activated carbon materials: dried date-palm leaf stalks (P_RS) and black alder cone-like flowers (A_RS). The sample of dried date-palm leaf stalk (*Phoenix dactylifera* L.) was collected in Nea Kalikratia, Greece. The sample of black alder cone-like flowers (*Alnus glutinosa* L.) was collected in Bijelo Polje, Montenegro. The selected precursors represent waste biomass, palm (which is a species of Mediterranean climate), and black alder (which is a species of temperate continental climate). In order to compare the characteristics of those new made materials, the commercial activated carbon material AquaSorb^®^ HSl (8 × 30 MESH) (C_AC) was also characterized.

### 2.2. Synthesis of Materials

#### 2.2.1. Carbonization Process

The carbonization process was performed in a horizontal stainless steel tube furnace with a programmer. The carbonization process was performed in a flow of N_2_ atmosphere (50 cm^3^ min^−1^). The furnace temperature was increased from room temperature to the desired operating temperature of 750 °C. The material was held at the operating temperature for 1 h. The flow of N_2_ gas in the furnace was maintained during cooling. The heating rate of the furnace was constant at 4 °C/min. Carbonated samples were labelled P_CC and A_CC for dried date-palm leaf stalk and black alder cone-like flowers, respectively.

#### 2.2.2. Activation Process

The activation of material was performed in a horizontal furnace with a programmer. For the activation process, firstly, the N_2_ was injected to flow in the system until the temperature reached 750 °C (50 cm^3^ min^−1^). When the temperature reached the desired operating temperature, it was held for 1 h in an atmosphere of CO_2_ (50 cm^3^ min^−1^). After a set time, the N_2_ was released again until the furnace had cooled to room temperature. The activated carbon samples were labelled P_AC and A_AC.

The calculation of the percentage loss of raw sample mass during the burn-off process was based on the yield of the activated carbon material. The mass-yield percentage was determined by formula:

Y = m/m_0_ × 100, (1)

where is Y—yield of activated carbon material, m—final mass of the sample after activation process, and m_0_—the initial mass of the raw sample.

The percentages of mass loss of the raw samples during the burn-off after the carbonization stage were 47.0% for the date-palm leaf stalk and 38.2% for the black alder cone-like flowers. The carbonization stage is necessary in order to convert organic lignin and cellulose into carbonaceous materials, with a reduction of the water, oxygen, hydrogen, sulfur, and other elements. Further, after the activation process, the total percentages of the loss during the burn-off of the raw samples were 63.5% for date-palm leaf stalk and 75.3% for black alder cone-like flowers. After the activation process, the obtained values were 36.5% for the date-palm leaf stalk and 24.7% for the black alder cone-like flowers.

### 2.3. Characterization of Materials

The physical and chemical characterizations of the material were performed on the raw samples (RS) after the process of carbonization (CC) and activation (AC). The physical–chemical characteristics of the raw samples and the carbonized and activated carbons were analyzed in order to consider all parameters for future use.

#### 2.3.1. Proximate/Ultimate Analysis and Lignocellulose Composition of Raw Samples

The proximate/ultimate analysis and lignocellulose composition were performed on the raw samples. In accordance with the TAPPI standard T 257 cm-12 method, the mechanically ground material was ground in a laboratory micro-mill with hammers. After sieving, 0.4–1 mm particles were taken for chemical composition analysis. The moisture content in the tested samples was determined gravimetrically, according to the standard TAPPI method T 264 cm-97; the cellulose content in the samples was determined by the Kurschner–Hoffer method [22]; and the lignin content after extraction (toluene-ethanol) was determined by the Klason method (T 222 om-11). To determine the content of the extracts that was soluble in organic solvents, according to the standard TAPPI method T 264 cm-97, a mixture of toluene and ethanol in a volume ratio of 2:1 (C_6_H_5_CH_3_/C_2_H_5_OH = 2/1, *v/v*) was used. The content of the extractive substances soluble in hot water was determined according to the TAPPI method T 207 cm-99 (also ASTM D1110-84), and the content of the mineral substances was determined through ash according to the standard method T 413 om-93 at 900 °C. The hemicellulose content was determined approximately, in addition to the content of certain components up to 100%.

#### 2.3.2. X-ray Powder Diffraction (XRD)

Commercial activated carbon, raw, carbonized, and activated samples of black alder cone-like flowers, and the dried palm leaf were analyzed by X-ray powder diffraction (XRD), which was performed using Rigaku (Rigaku International Corporation, Tokyo, Japan) Ultima IV equipment using Ni-filtered Cu Kα radiation and the step-scan mode (2*θ* -range: 5° to 90° in a continuous step with a scan mode width of 0.02° and 0.5° min^−1^). The software package Powder Cell [23,24] was used to identify the phases present in the samples. The TCH pseudo-Voigt profile function gave the best fit to the experimental X-ray data. All the structure models for refinement, i.e., cif files, were taken from the American Mineralogist Crystal Data Structure Base (AMCDSB) [25] and the Cambridge Crystallographic Data Centre CCDC.

#### 2.3.3. Fourier-Transform Infrared Spectroscopy (FTIR)

Samples of commercial activated carbon, raw samples, and carbonized and activated samples of black alder cone-like flowers and the dried palm leaves were analyzed by Fourier-transform infrared spectroscopic analysis (FTIR). FTIR spectra were performed on a Thermo Scientific Nicolet iS50 instrument. The spectra were recorded in transmission mode in the range of 4000–450 cm^−1^. The resolution and number of scans were 2 cm^−1^ and 64, respectively. Two corrections—automatic correction of base line and atmosphere suppression—were used after recording the spectra.

#### 2.3.4. Nitrogen Physisorption at −196 °C

The textural characteristics of the commercial activated carbon (C_AC) and the activated samples of palm leaf stalk (P_AC) and black alder cone-like flowers (A_AC) were determined through nitrogen physisorption at −196 °C using a Sorptomatic 1990 Thermo Finnigan. Before measurement, the sample was degassed for 4 h at room temperature and for 18 h at 350 °C under vacuum. The resulting N_2_ isotherms were analyzed by Software ADP Version 5.17 CE Instruments. The specific surface area (S_BET_) was calculated using the BET (Brunauer, Emmet, Teller) equation according to the recommendations for carbon materials [26]. To determine the microporosity, several methods were applied, including the Dubininee–Radushkevich [27], Horvathe–Kawazoe (HK) [28], and t-plot methods. The volume of the mesopores was determined from both branches (adsorption and desorption) for the part of the relative pressure that corresponds to the range of the diameters of the mesopores (2–50 nm).

#### 2.3.5. Field Emission Scanning Electron Microscopy (FESEM)

FESEM measurements were carried out before and after the activation of the samples. To examine the surface morphology of the samples, field emission scanning electron microscopy (FE-SEM) and the TESCAN Mira3XMU instrument at 20 kV (Tescan, Brno–Kohoutovice, Czech Republic) were used. Before analyses were conducted, the samples were sputter-coated with Au/Pd alloy, using the sputter coater Polaron SC503 Fision Instrument (Quorum Technologies Ltd., Lewes, UK).

### 2.4. Qualitative and Quantitative Phytoplankton Analysis

The application of the materials was tested on water samples from a highly eutrophic lake from Southern part of Serbia, Aleksandrovac Lake. In the time period of January 2020–December 2021, water quality checks were conducted monthly at Aleksandrovac Lake. Sampling was carried out from a boat, and water samples were taken from a depth of 1 m with a Ruttner sampler for quantitative phytoplankton analysis. A plankton net (mesh size 22–23 μm, net frame 25 cm ø) was used for collecting the samples for qualitative phytoplankton analysis. All samples were immediately fixed in 4% formaldehyde. The samples were transported within a short time interval and, within 24 h of the sampling process, processed to the laboratory.

The abundance of phytoplankton (number of cells/mL) was evaluated with a Zeiss Axio Observer.Z1 inverted microscope, according to the Utermohl method (SRPS EN 15204: 2008). Standard keys were used for the identification of phytoplankton species [29,30,31,32,33,34,35,36,37], while the classification presented by Reynolds, 2006 [38], was used to group species into several divisions: Cyanobacteria, Chlorophyta, Euglenophyta, Cryptophyta, Xanthophyta, Chrysophyta, Bacillariophyta, and Dinophyta.

### 2.5. Analysis of Adsorption Properties of Material

The adsorption properties of the material were analyzed for the water sample from the shallow lake with the high eutrophication and with the presence of “water bloom”. The parameter that was followed was the number of cyanobacterial cells before and after the treatment with activated carbon materials. Four samples of 25 mL were separated from the same water sample. One of them was raw water, where the number of cyanobacterial cells before treatment was analyzed. A total of 0.25 g of the materials was placed into each of the other three samples. The solution with activated carbon was mixed using a mechanical stirrer at a speed of 110 rpm for 24 h. The samples were taken from a mechanical stirrer filtered through slow filter paper. Quantitative cyanobacteria analysis (number of cells/colonies/filaments) was performed by the standard method (SRPS EN 15204: 2008) by Utermöhl (1958) at the Institute of Public Health of Serbia “Dr Milan Jovanović Batut”, using a Zeiss Axio Observer.Z1 invert light microscope.

This method is used for the assessment of the absolute number of organisms that are temporarily deposited in sedimentation chambers. The removal efficiency of active carbon (%),is estimated by the following equation:

RE = ((Ni − Nt))/Ni ×100,(2)

where RE is removal efficiency, Ni the initial number of cyanobacterial cells, and Nt is the number of cyanobacterial cells at time t (after 24 h).

## 3. Results and Discussion

### 3.1. Results of Physical and Chemical Characterization of Materials

#### 3.1.1. Lignocellulose Composition

Results of lignocellulose composition of raw samples of dried date-palm leaf stalks (P_RS) and black alder cone-like flowers (A_RS) are presented in Table 1. The results are expressed in relation to the absolutely dry masses of the material (wt.%, dry) and are presented as the mean values (arithmetic mean) of the three repeated versions.

The average lignocelluloses biomass contains an average 10–30% of lignin [8]. In the raw samples of date-palm leaf stalks, the lignin content is very low (7.9%), and for black alder cone-like flowers it is very high (29.6%). The high content of extracts in the hot water of both samples, especially in the date-palm leaf stalk (48.66%), is probably due to the solubility of some hemicelluloses in water [11]. Therefore, the hemicellulose content obtained is calculated to be low. The results for palm tree leaves are different for comparing to those found in the study by Bendahou et al., 2007 and Alotaibi et al., 2019 [11,12], and are generally lower. The difference for lignocellulose biomass content in date-palm leaf stalks depends on soil differences and the climatic conditions in which the date palm trees growth occurs [12]. Namely, the proportions of cellulose, hemicelluloses, and lignin at the end of the carbonization phase and the structural rearrangement of carbon may also play an important role in the adsorption properties of the final activated carbon amount [39]. The microstructures of activated carbons depend not only on the experimental conditions of the carbonization and activation steps, but also on the original lignocellulosic raw material, that is, on the lignin, hemicelluloses, and cellulose content [40]. The cellulose activation can produce a mixture of pore size carbons, whereas in activation of lignin carbons which are mostly microporous are produced [40]. Moreover, the surface and porosity of the carbon materials produced with a high lignin content are higher compared to activated carbons from materials with a lower lignin content [40].

The results for black alder cone-like flowers are quite the same as in the study of Mokrzycki et al., 2020 [13]. The similarity is due to the fact that they grow in very similar climatic conditions. The black alder cone-like flowers represent the better precursor compared to date-palm leaf stalks, due to their higher content of lignocelluloses.

#### 3.1.2. Results of X-ray Powder Diffraction

**Figure 2:**
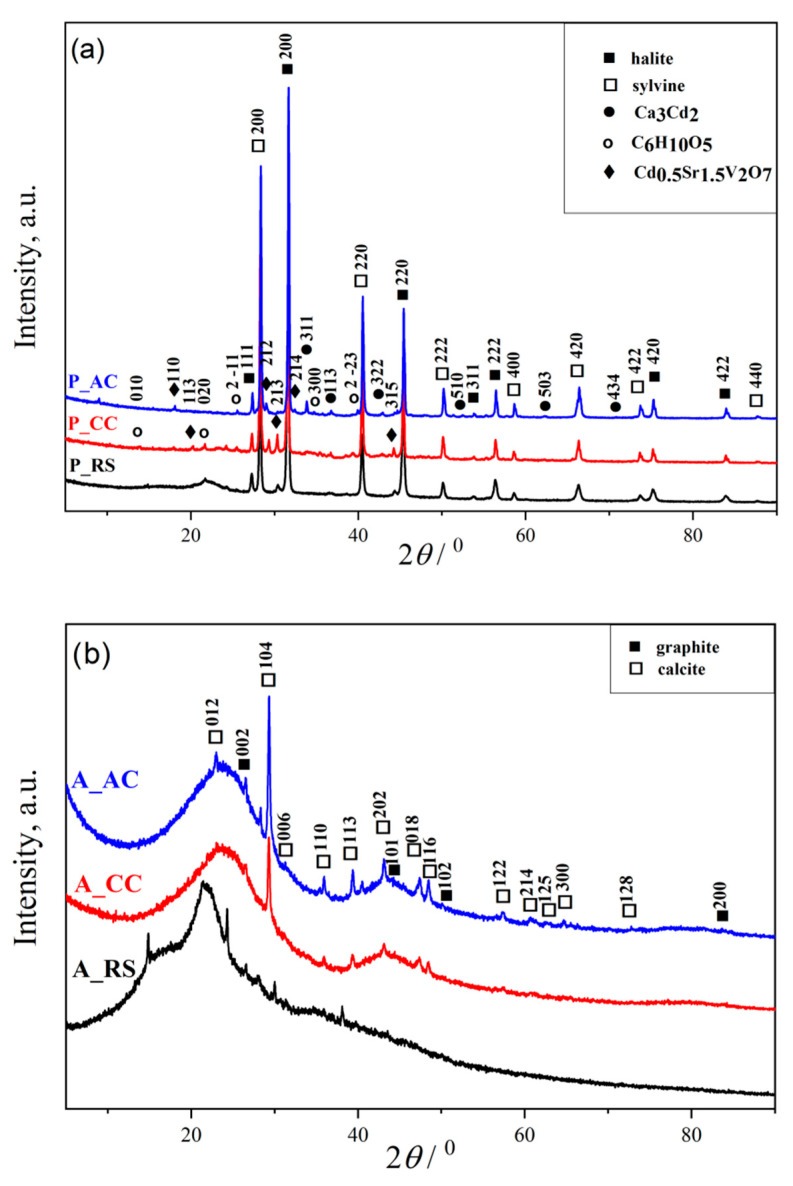

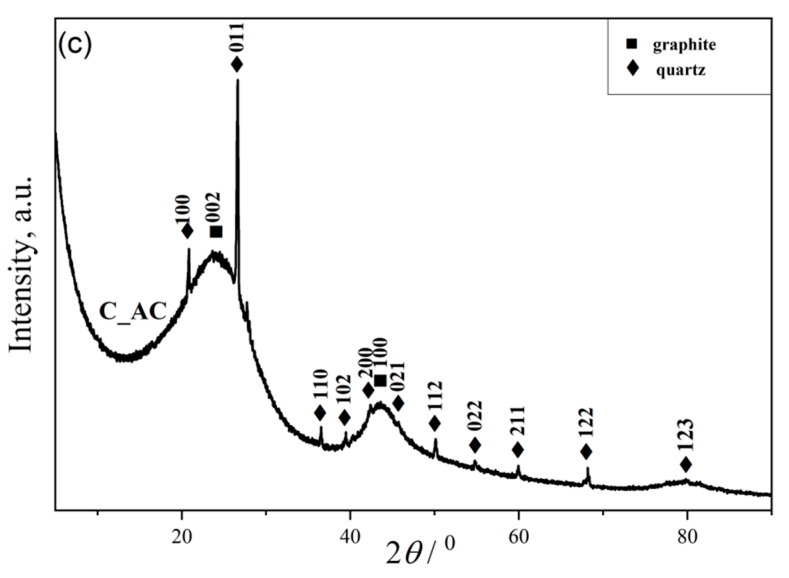
XRD spectra of the: (**a**) raw (P_RS), carbonized (P_CC), and activated (P_AC) dried palm leaf stalk sample; (**b**) raw (A_RS), carbonized (A_CC) and activated (A_AC) black alder cone-like flowers; (**c**) commercial activated carbon (C_AC).

The X-ray patterns of dried palm leaf stems (raw (P_RS), carbonized (P_CC), and activated (P_AC)) shown in Figure 2a indicate the multiphase composition of all three materials. All narrow peaks with high intensities belong to minerals halite (sodium chloride, ICSD No. 60280) and sylvine (potassium chloride, ICSD No. 154214). Other low intensities peaks belong to Ca_3_Cd_2_ (calcium cadmium, ICSD No. 30082) and Cd_0.5_Sr_1.5_V_2_O_7_ (strontium cadmium divanadate, ICSD No. 39806). The powder diffractograms did not show all Miller indices for graphite (carbon, ICSD No. 617290) and cellulose (C6H10O5, CSD No. JINROO01), due to the overlap of their reflections. By analyzing the XRD spectrum, it was observed that the most represented peaks belonged to the minerals halite and sylvin [12]. We believe that this is a consequence of sampling palm leaves in the coastal area where the marine process is pronounced. Moreover, the presence of Ca_3_Cd_2_ and Cd0.5Sr1.5V2O7 directs us to the region from which the material was sampled, so their presence is expected [41,42,43]. The peaks, which correspond to the presence of a cellulose crystalline structure, are observed at 13.806°, 21.799°, and 25.799° attributed to the cellulose content [44].

The XRD patterns of the powders obtained from the black alder cone-like flowers (raw (A_RS), carbonized (A_CC), and activated (A_AC)) are shown at the Figure 2b. From Figure 2b, it can be noticed that the presence of an amorphous phase in all samples is significantly higher than in the samples shown in Figure 2a. The XRD patterns reflect the presence of two phases of graphite and calcite in all raw, carbonized, and activated black alder cone-like flowers samples. First, the diffraction peaks located at 25.689° and 42.214° are weak and stretched, which indicates a small grain size, and the grain shape was not complete; further, they belong to the graphite with Miller indices (002) and (100), respectively (ICSD No. 617290). Wide-ranging peaks from graphite suggest a weak crystalline structure [45], indicate the presence of a graphite-type carbon, considered to be as turbostratic graphitic carbon [46,47]. Second, there are narrow peaks with low intensities, which are characteristic of the presence of CaCO_3_ (calcium carbonate; calcite; ICSD No. 158258) and designate its good crystallization of structure and small particle size. Calcium-carbonate is usually present in plants, which explains its presence in raw samples (A_RS). On the other hand, the increase in the intensity of peaks is characteristic for calcite in A_CC and A_AC compared to the raw sample, which is a consequence of carbonization and activation due to the pyrolysis process of the carbon material from biomass that leads to better ordering of the crystal lattice of calcite [48,49].

For the purpose of comparison, we used acid-washed coconut-based activated carbon (C_AC) (Figure 2c). The broad and weak diffraction peaks belong to graphite. Moreover, there are several peaks with conspicuous intensities, which are characteristic of the presence of SiO_2_ (silicon oxide, ICSD No. 174). Based on the XRD analysis (Figure 2c), it was determined that quartz was also present in the commercial sample, in addition to the required activated carbon.

#### 3.1.3. Results of Fourier-Transform Infrared Spectroscopy

**Figure 3:**
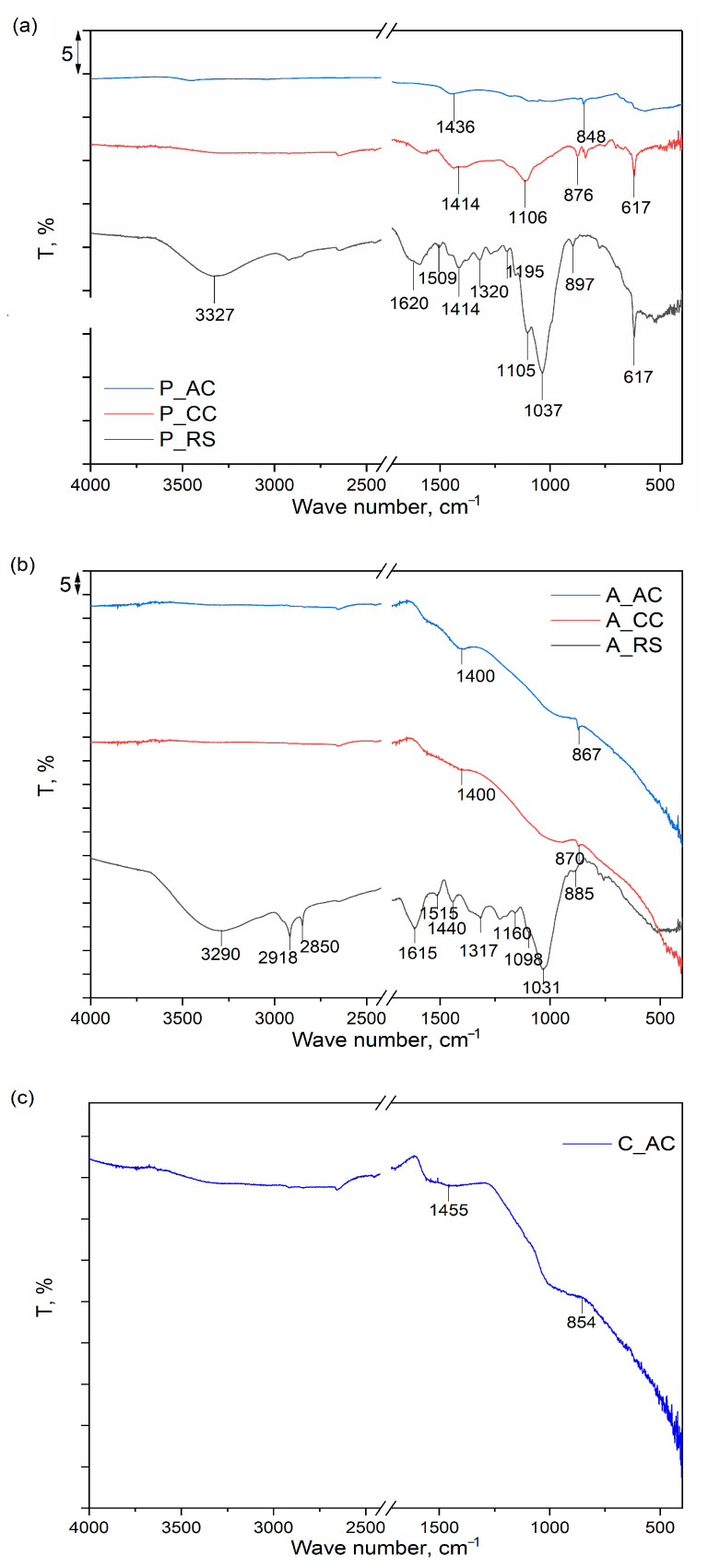
FTIR spectra of the: (**a**) raw (P_RS), carbonized (P_CC), and activated (P_AC) dried palm leaf stalk sample; (**b**) raw (A_RS), carbonized (A_CC), and activated (A_AC) black alder cone-like flowers; (**c**) commercial activated carbon (C_AC).

The results of the FTIR analyses of the samples of dried palm leaf stalk (P) and black alder cone-like flowers (A) in carbonized (P_CC and A_CC) and activated forms (P_ and A_AC), as well as FTIR spectrum of the commercial activated carbon sample (C_AC), are provided in Figure 3a–c.

As can be seen from Figure 3a,b, both raw samples, P_RS and A_RS, have very similar FTIR spectra, in which there are dominant bands characteristic of the main constituents (cellulose, lignin and hemicellulose) detected by proximate/ultimate analysis (Table 1). For the P_RS, the positions of the spectral bands that are characteristic of lignin are: 1620 and 1509 cm^−1^, which originated from aromatic skeletal vibrations; 1414 cm^−1^ due to C-H deformation; and 1320 cm^−1^, characteristic of syringyl ring plus guaiacyl ring vibrations [50]. For the A_RS, the same spectral bands were also detected but were slightly shifted in positions: 1615, 1515, 1440, and 1317 cm^−1^. For cellulose in the sample, P_RS is visible in spectral band at 3327 cm^−1^, which is characteristic for the stretching vibration of O-H bonds in polysaccharides. The peak is characteristic of the stretching vibration of the hydroxyl group in polysaccharides and also includes inter- and intra-molecular hydrogen bond vibrations in cellulose. The band at 1105 cm^−1^ can be assigned to the glucose ring stretching vibration, while the band at 897 cm^−1^ can be attributed to vibrations in the amorphous region in cellulose. For cellulose, there are also characteristic bands at 1620 and 1414 cm^−1^, which overlap with lignite bands and originated from the vibration of water molecules absorbed in cellulose and vibrations in the crystalline structure of the cellulose, respectively [51,52]. For the sample A_RS, the same spectral bands were detected for cellulose but slightly shifted in positions: 3290, 1615, 1440, 1098, and 885 cm^−1^. Moreover, for the sample A_RS, two additional spectral bands at 2918 and 2850 cm^−1^ are visible, which can be attributed to CH stretching vibrations in the cellulose [51]. The spectrum of the hemicellulose is similar to the spectrum of cellulose, because of their structural resemblance, and almost all spectral bands characteristic of hemicellulose overlap with those that originated from cellulose. The spectral bands at 1037 cm^−1^ for P_RS and 1031 cm^−1^ for A_RS are only proof of the presence of hemicellulose and are assigned to C–O, C–C stretching, or C–OH bending in xylan [53].

The spectrum of NaCl detected by the XRD in the sample P_RS is characterized by bands at about 1100 cm^−1^ and 1600 cm^−1^ [54]^.^ However, these bands were not detected in the spectrum of the P_RS, due to them overlapping with other spectral bands. On the other hand, KCl and graphite do not possess characteristic spectral bands in the investigated range of wave numbers, so they could not be detected in the FTIR spectra of the raw samples of P_RS and A_RS [55,56].

After the thermal treatment at 750 °C of both samples (P_CC and A_CC), it is noticeable that there is a significant loss of spectral bands, and only a few bands derived from cellulose and/or lignin are visible that are simultaneously significantly reduced in their intensity, which is a clear indication of a successful carbonization process. After additional thermal treatment and activation with CO_2_ (P_AC and A_AC), due to the reaction of the carbon with CO_2_, a further decrease in the spectral bands’ intensities was detected for both samples, and only bands in the regions 1400–1450 cm^−1^, which originated from the presence and vibrations of aromatic C=C groups and the 840–870 cm^−1^ bending vibration of the C–H bonds in the high degree of substitution of the aromatic rings, were detected. The similarity of the spectra of carbonized and physically activated samples for both dried palm leaf stalks and black alder cone-like flowers with the spectrum of chemically activated commercial activated carbon [57,58] indicates that the method of activation (physical or chemical) does not significantly affect the structural properties of these materials but is mostly affected by the activation temperature.

#### 3.1.4. Results of Low Temperature N_2_ Physisorption

**Figure 4:**
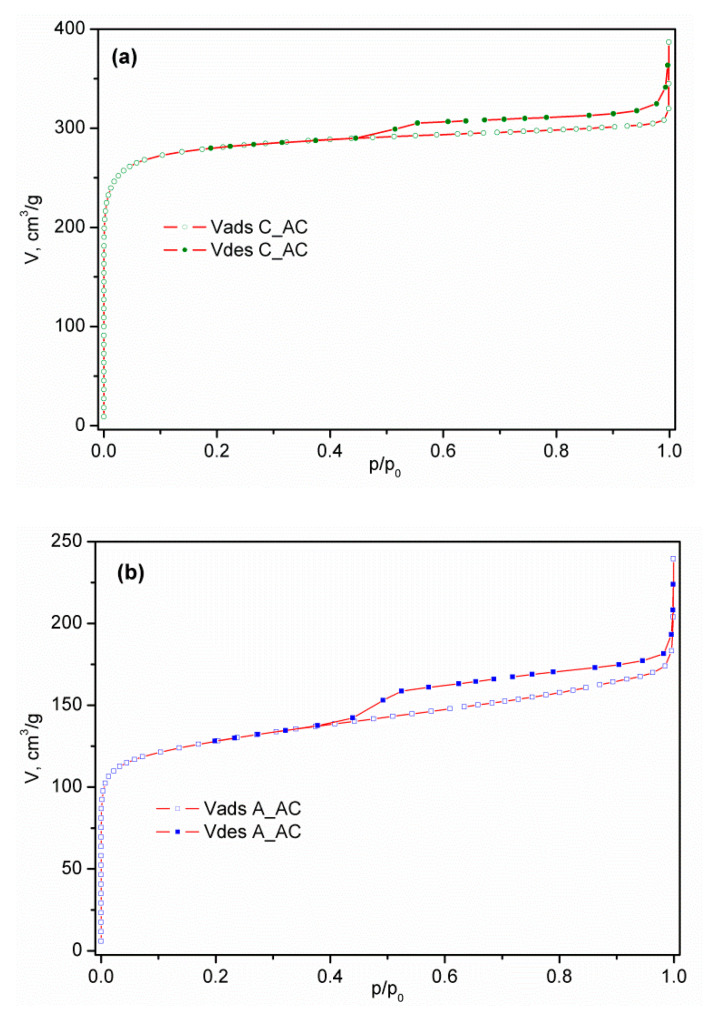

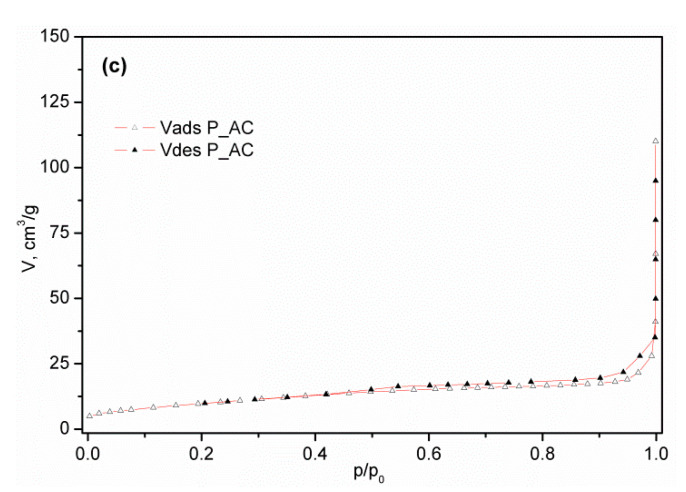
N_2_ adsorption–desorption isotherm of activated samples: (**a**) C_AC, (**b**) A_AC, and (**c**) P_AC.

The nitrogen adsorption/desorption isotherms of commercial activated carbon (C_AC) and activated samples of palm leaf stalk (P_AC) and black alder cone-like flower (A_AC) measured at −196 °C are shown in Figure 4, while the corresponding calculated textural parameters are presented in Table 2. Having in mind IUPAC classification [26], the isotherm of commercial activated carbon presented in Figure 1a is similar to the N_2_ physisorption isotherm type I, characteristic of microporous samples. The high adsorption volume in the p/p_0_ < 0.1 region confirms the highly developed microporosity, such as in many other carbons [59,60,61,62,63]. The activated carbon isotherm obtained from the conical flowers of black alder, shown in Figure 1b, has a mixed character. Indeed, in addition to the obvious contribution of the microporous part in the region of small p/p_0_ values, the clear presence of the isothermal slope in the region of relative pressures above 0.2 and the existence of a hysteresis loop indicates the existence of a mesoporous system in the material. The absence of a horizontal plateau in the region of higher values of relative pressures excludes the type IV isotherm, so the resulting form of the isotherm of A_AC material is type I modified by the contribution of the properties which are characteristic of type II (macro-porous or nonporous materials). On the other hand, the activated carbon isotherm from the palm leaf stalk shown in Figure 1c belongs to type II, which, together with the relatively small values of V_tot_ and S_BET_, reveals the non-porous nature of P_AC. In Table S1 presented in Supplementary Materials S2, the ratios V_mic_/V_meso_ for the commercial sample (C_AC) and for the volume of mesopores calculated from the adsorption branch are in the range of 7.4–8.0, while for V_mezo_ this ratio is in range of 4.4–4.3. This shows undoubted microporous properties. On the other hand, for the A_AC materials, this ratio is in the range of 1.9 to 2.5 when using V_meso_ (ads) and 1.5 to 1.9 when using V_meso_ from the desorption branch. It is clear that the contribution of mesopores to the total pore volume is incomparably more significant in the A_AC sample.

The hysteresis in the region of the relative pressure range above 0.4 is otherwise not uncommon; in fact, it expectedly occurs in most of the materials characterized by nitrogen physisorption. The existence of the loop in this region indicates the presence of a pore system inside the particles (intra-particle porosity) and/or voids between the weakly bound particle agglomerates (inter-particle porosity).

Analyzing the results for commercial active carbon, it is not surprising that the obtained value of S_BET_ exceeds 1100 m^2^ g^−1^. However, the value of the specific surface area calculated by the BET equation can sometimes be significantly overestimated, because capillary condensation in micropores can begin before the complete formation of the N_2_ monolayer, leading to the presentation of an S_BET_ value even higher than the theoretical one. Therefore, we used a t-plot and found that there is no significant deviation in the value of the specific surface area. This means that the amount of nitrogen packed within an adsorbed monolayer is the same as estimated by the BET equation. In addition, since various methods (t-plot methods, DR-method, and HK method) produced mutually comparable high values of micropore volume of commercial activated carbon, in the range of 0.42–0.46 cm^3^ g^−1^, we concluded that the sample is dominantly microporous.

However, analyzing the results for activated carbon obtained from bio-waste palm leaf stalks and black alder cone-like flowers, the situation is somewhat different. The obtained value of S_BET_ for activated carbons obtained from bio-waste palm leaf stalks is 36.6 m^2^ g^−1^, while for activated carbons obtained from bio-waste black alder cone-like flowers the value is 485 m^2^ g^−1^. As can be seen, these are samples of completely different textural properties.

Sample A_AC has a value of S_BET_ which is more than twice less than the value of the value for sample C_AC. However, comparing the value of specific surface area for A_AC determined by t-plot, we found that there is no significant deviation in the values of the specific surface areas obtained using the BET method. What is interesting is that this sample shows the presence of mesopores, in addition to micropores, in a certain percentage. Namely, according to the results shown in Table 2, the sample has a certain mesoporous surface, which is 65% larger than the surface of the mesopore of commercial C_AC (10.4 m^2^ g^−1^ vs. 6.3 m^2^ g^−1^). Applying various methods (DR-method and HK method) obtained mutually comparable values of micropore volume in the range of 0.19–0.21 cm^3^ g^−1^, while the volume of mesopore determined by the BJH and DH methods were 0.097 cm^3^ g^−1^ and 0.125 cm^3^ g^−1^, respectively. Additionally, comparing the V_mic_ values for C_AC and A_AC, the values for active carbon were clearly between 1.9 and 2.3 times higher, depending on the model e.g., V_mic_HK is 0.431 cm^3^ g^−1^ vs. 0.195 cm^3^ g^−1^ in favor of C_AC. Not only that, the corresponding absolute values of the intermediate commercial sample are less than the values for the sample of A_AC. This is the decisive difference, in terms of the total contribution of the individual pore types to the general textural properties of the material (which is clearly seen through the differences in the slope of the isotherms in the previously mentioned region, p/p_0_).

#### 3.1.5. Results Field Emission Scanning Electron Microscopy

**Figure 5:**
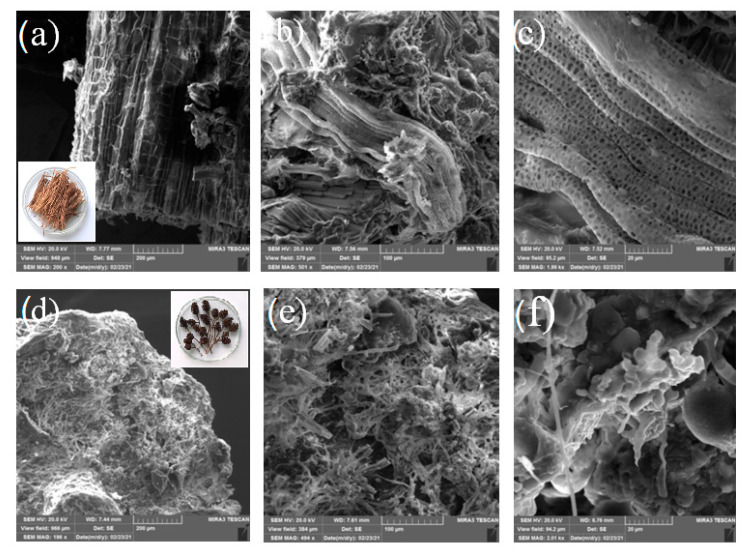
FESEM micrographs at different magnifications of P_RS (**a**) 200× (inset: visual appearance), (**b**) 500×, and (**c**) 2000×; and A_RS (**d**) 200× (inset: visual appearance) and (**e**) 500×, (**f**) 2000×.

In Figure 5 the FE-SEM micrographs of raw samples of palm leaf (Figure 5a–c) and common alder (Figure 5d–f) at different magnifications (200, 500 and 2000×) are presented. As can be seen, significant differences are visible in the morphological properties of the investigated samples. For the sample of P_RS, the dominant presence of the fibrous structures is visible. In the literature, such structures are described for samples where cellulose is dominant in their structures, with a certain percentage of hemicelluloses [64,65]. This is expected for this sample, since the results of the determination of the lignocellulose composition (Table 1) showed a dominant presence of the cellulose with hemicelluloses (26% in total).

For A_RS samples (Figure 5d–f), a significant different morphology was visible. On the surface of this sample, in addition to fibrous structures characteristic of cellulose and hemicellulose, microspheres and microplates are also present to a significant extent and homogenously dispersed on the observed area of the surface. According to the literature [66], such nonfibrous structures are characteristic for lignin, and the morphology can be explained by the significantly higher content of the lignin (30%; Table 1) in this sample, which was equal to the content of the cellulose and hemicelluloses (30% in total; Table 1). The structure characteristic of lignin was not dominantly visible in the sample raw palm leaf due to the significant lower lignin content in that sample (7.9%, Table 1).

**Figure 6:**
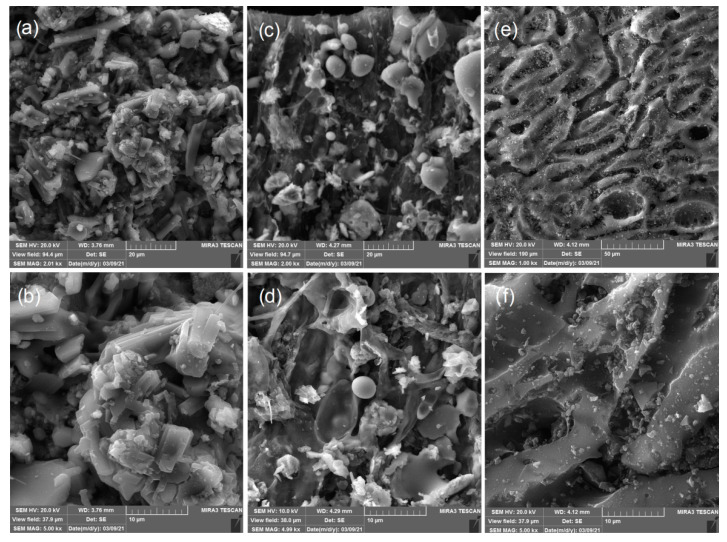
FESEM micrographs at different magnifications of P_AC (**a**) 2000× and (**b**) 5000×; and A_AC (**c**) 2000× and (**d**) 5000×; and C_AC (**e**) 1000× and (**f**) 5000×.

The raw samples of P_RS and A_RS were changed to activated carbon materials P_AC and A_AC by carbonization and activation processes. The FE-SEM micrographs of the activated carbon materials and commercial activated carbon are presented in Figure 6. From Figure 6a–d it is clear that, after the carbonization and activation process, the morphologies of both the palm leaf and the common alder were significantly changed in comparison with the raw samples. This was the case namely due to the carbonization; the organic lignin and cellulose were converted into carbonaceous materials. As a consequence of that process, a reduction in the amount of water, oxygen, hydrogen, sulfur, and other elements occurs, and such reactions facilitate subsequent activation reactions [67]. After the process of physical activation with CO_2_ on the surfaces of both activated carbons, irregularly distributed plates of different shapes are visible, between which channels and cavities of different sizes and shapes can be observed. Literature data [68] showed that CO_2_ enables the formation of a hierarchical porous structure with aromaticity of these samples. In addition, the literature data [68] also showed a possible change of the polarity and acidity of the carbon surface, which provide higher densities of functional groups near the surface of the activated carbon. The formation of functional groups near the surface of the activated carbon is consistent with the FTIR results for both samples and indicates to us the possibility of further interpreting the detailed mechanism of removing cyanobacteria.

On the other hand, as can be seen in Figure 6e,f, the surface of the commercial activated carbon is smooth has well-distributed pores and cavity structures. The small particles that can be seen on the entire surface of the sample of commercial activated carbon (as a scattered powder) are characteristic of the process of chemical activation, which is well described in the literature [69]. It is also clear from Figure 6a–d that the surfaces of the activated carbons from the palm leaf and common alder that were obtained by physical activation differ significantly from the surface of the commercial activated carbon, indicating that commercial activated carbon is obtained by chemical activation.

### 3.2. Results of Qualitative and Quantitative Phytoplankton Analysis

#### 3.2.1. Results of Qualitative Phytoplankton Analysis

Changes in water quality are reflected in changes in the composition of the phytoplankton community. With the increase of eutrophication, there are changes in the representation of the different phylum of algae and, therefore, an increase in Cyanobacteria. The monitoring of the Aleksandrovac Lake water quality was conducted in order to evaluate its ecological potential since 2008. The presence of Cyanobacteria, including the invasive tropical and potentially toxic species Raphidiopsis raciborskii, indicates the low quality of the water and the possible harmful effects on human health.

**Figure 7:**
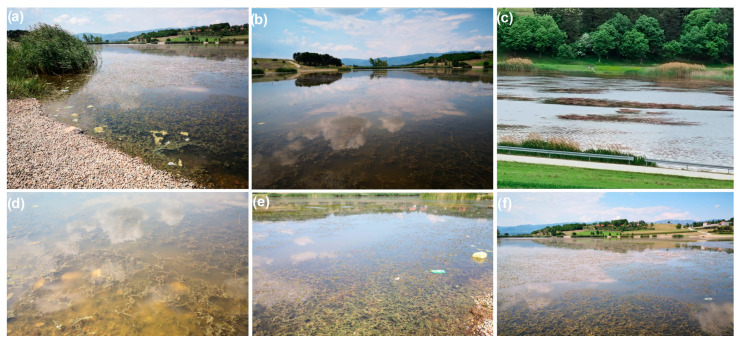
Visually determined the coverage of the lake by the submerged macrophytes: (**a**–**c**) observation of the whole Lake; (**d**–**f**) observation of the coastal part of the Lake.

In the period from January 2020–December 2021, monthly water quality checks of the Aleksandrovac lake were conducted. The samples were transported in a short time interval, and, within 24 h of the sampling process, they were processed in the laboratory. On site, it was visually determined that the coverage of the lake by the submerged macrophytes is almost 80% whenever the field is entered, as evidenced by Figure 7a–f in this paper.

The appearance of the Aleksandrovac lake indicates a baffling ecosystem with highly developed submerged vegetation (dominated by *Miriophylum* sp.). Moreover, emergent macrophytes, *Typha* sp. and *Phragmites* sp., are well developed on the rim of the water body. On the basis of field visits and the situation at the lake, it can be immediately seen that the Aleksandrovac Lake will receive a bad grade, because the hydrological regime is extremely bad due to the low water level during summer and greater evaporation than precipitation.

The phytoplankton community composition is strongly influenced by changes in the water quality and the tropic state of the waterbody. As the eutrophication process increases, the percentage of different phytoplankton groups alters. In this regard, it has been noted that the abundance of cyanobacteria and green algae increases along the trophic gradient [20].

Assessing the quantitative phytoplankton community composition aims to determine the spatial and temporal phytoplankton dynamics. The abundance of organisms (both cells and colonies) provides very useful information about the populations of different species. This type of analysis is particularly important in monitoring seasonal changes of phytoplankton species [20]. Additional phytoplankton monitoring was implemented in this study to establish their seasonal qualitative and quantitative trends during the period from January 2020–December 2021.

**Figure 8:**
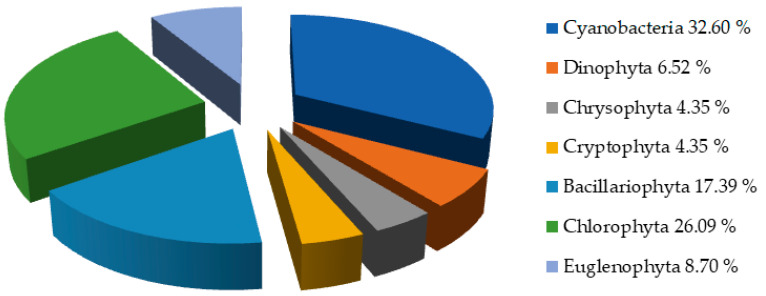
Qualitative analyses of phytoplankton from January to December 2021.

Qualitative analysis of phytoplankton in all seasons revealed the presence of 46 taxa (Table 3) from 7 phyla (*Cyanobacteria, Dinophyta, Chrysophyta, Cryptophyta, Bacillariophyta, Chlorophyta*, and *Euglenophyta*). In this regard, according to the obtained results, it can be concluded that Cyanobacteria had the highest abundance within the phytoplankton community (Table 3 and Figure 8). The highest phytoplankton diversity was recorded in August, while the lowest diversity was observed in February. The percentage of different phytoplankton taxonomical groups identified in Aleksandrovac Lake is presented in Figure 8. Cyanobacteria showed the highest species diversity (32.60% of total identified taxa), although the number of species of Chlorophyta was also relatively high (26.09%).

It is known that different cyanotoxins can be released as a consequence of cyanobacterial bloom. In order to assess the potential danger of the produced toxins based on the literature data, the toxins that could be produced by detected cyanobacteria in the period from January 2020 to December 2021 are presented in Table 4. Additionally, in order to assess the effects on animal and human health, the chemical structure, molecular weight, and effect with negative impact when using contaminated water are presented in Table 5.

#### 3.2.2. Results of Quantitative Phytoplankton Analysis

**Figure 9:**
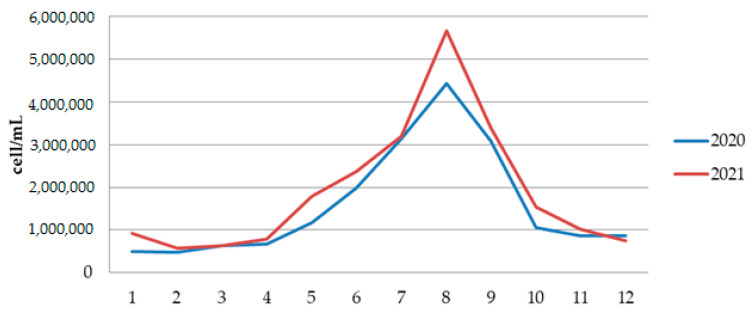
The phytoplankton abundance in Aleksandrovac Lake during 12 months in 2020 and 2021.

Qualitative analysis of phytoplankton for two years indicates a high diversity of both Cyanobacteria and Eukaryotic algae. The total number of phytoplankton cells in 2020 ranged from a minimum of 468,486 cells/mL, in February, to a maximum of 4,430,948 cells/mL in August 2020 (Figure 9 and Table S2). The total number of phytoplankton cells in 2021 ranged from a minimum of 578,631 cells/mL, in February, to a maximum of almost 5,657,658 cells/mL in August 2021 (Figure 9 and Table S3).

**Figure 10:**
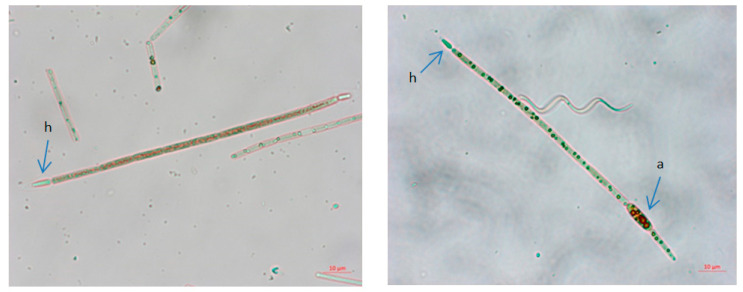
Photomicrographs of *Raphidiopsis raciborskii* from Aleksandrovac Lake. Scale bars 10 µm. Legends: a—akinetes, h—heterocyst.

The abundance of cyanobacteria in Aleksandrovac Lake ranged from 462,156 cells/mL in February 2020 to 5,093,067 cells/mL in August 2021, which indicates very high human and animal health risk level (Tables S2 and S3). Qualitative analysis of phytoplankton confirmed the dominant presence of the invasive and potentially toxic Raphidiopsis raciborskii (Figure 10).

**Figure 11:**
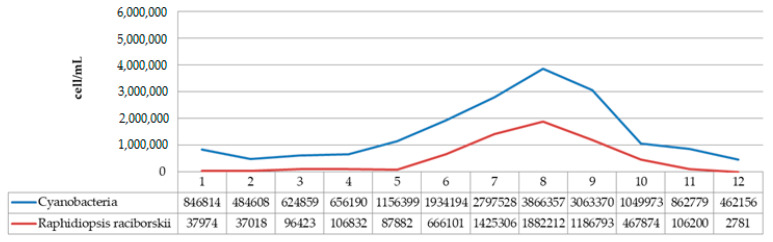
Dynamics of the average cell number of cyanobacteria compared to abundance of *R. raciborskii* from January 2020 to December 2020.

**Figure 12:**
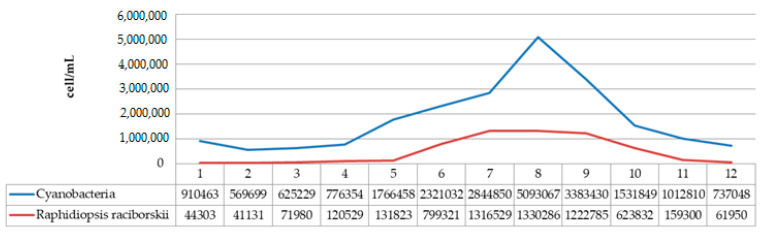
Dynamics of the average cell number of cyanobacteria compared to abundance of *R. raciborskii* from January 2021 to December 2021.

The species *R. raciborskii* was present in the phytoplankton community of Aleksandrovac Lake during the entire study period. The temporal variation of *R. raciborskii* abundance was observed both during 2020 and 2021, where minimums were recorded in February (2781 and 41,131 cells/mL, respectively) and maximums in August (1,882,212 and 1,330,286 cells/mL, respectively). Tables S2 and S3 show the dynamics of the average cyanobacteria cell numbers during the study period in 2020 and 2021. Additionally, Figure 11 and Figure 12 show the dynamics of the average cell number of cyanobacteria compared to the abundance of *R. raciborskii* from January 2020 to December 2021.

**Figure 13:**
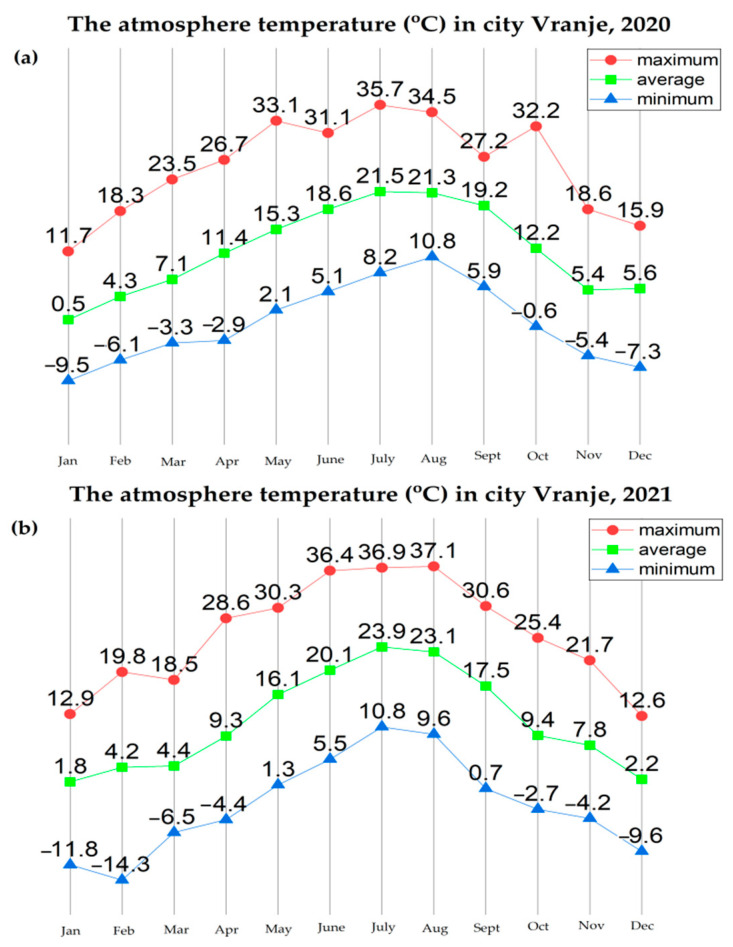
The atmosphere temperature in Vranje, in different months during: (**a**) 2020 and (**b**) 2021.

Further analysis of the environmental conditions and the obtained results showed that the total number of cyanobacterial cells was positively correlated with air temperature (Figure 13). Namely, it has been noticed that August represents the month with the maximum abundance of cyanobacteria during both years. In addition, the difference between the maximum values of cyanobacterial abundance in August 2020 (3,886,357 cells/mL) and August 2021 (5,093,067 cells/mL) was noticed, which is in line with the increase in the atmospheric temperature value in that month in both years (the difference between the values of the maximum atmospheric temperature was 2.5 °C; Figure 13).

Analysis of the obtained results also showed that the abundance of *R. raciborskii* increased during the warm months and decreased during the autumn and winter period of the year. In this study, the abundance of *R. raciborskii* was found to correlate with the atmosphere temperature, which is in accordance with the results obtained by Đorđević et al., 2015 [74], where the presence of higher temperature values was presented as the main factor responsible for the massive development of *R. raciborskii*. Additionally, during the investigation conducted in the Ponjavica River in 2008, Karadžić et al., 2013 [20], reported the additional peak of R. raciborskii abundance during the summer.

The abundance of *R. raciborskii* follows the trend, with the minimum of 2781 cells/mL in February 2020 when the average temperature was 4.3 °C, up to a maximum of 1,882,212 cells/mL in August 2020 when the average temperature was 21.3 °C (with the maximum recorded of 34.5 °C). In February 2021, the amount of *R. raciborskii* was measured at 41,131 cells/mL, at an average temperature of 4.3 °C, while the maximum value of R. raciborskii was 1,330,286 cells/mL in August 2021, at an average temperature of 23 °C, with the highest recorded of 37 °C. These results correlate with the studies Recknagel et al., 2014 [75] and Soares et al., 2013 [76], where blooms are described as being more likely to happen at the temperature of 25–32 °C, bearing in mind that this is considered to be a tropical species by its native areal. The widespread occurrence of *R. raciborskii*, that includes both tropical and moderate temperature regions, is possibly related to climate change conditions, which can cause an increase in the species areal. The total number of cyanobacterial cells indicates a very high level of risk to human and animal health, based on the recommendations given by the World Health Organization (WHO) [77]. Given that the species is potentially toxic, it is important to monitor its distribution and whether it forms blooms.

#### 3.2.3. Qualitative and Quantitative Analysis of Cyanobacteria from Aleksandrovac Lake before and after the Water Treatment

The World Health Organization (WHO 2003) [77] suggested three alert levels in relation to the potential health risks based on the abundance and density of cyanobacteria in recreational waterbodies. Accordingly, the emphasis of the preliminary research was on determining the adsorption properties for Cyanobacteria cell removal from contaminated water taken from Aleksandrovac Lake. After two years of monitoring Aleksandrovac Lake, it was determined that the peak of cyanobacterial abundance was recorded in August in both years. Accordingly, the preliminary research was conducted on a water sample from Aleksandrovac Lake from August 2021, and the results are presented in Table S3. The number of dominant Cyanobacteria individuals and the number of cells per mL of water before and after treatment with different activated carbons for 24 h are shown in Table S3.

The removal efficiency of activated carbons after 24 h for all three materials showed a high degree of adsorption of Cyanobacteria. The obtained results showed that the newly synthesized materials P_AC and A_AC are significantly effective for water treatment under applied experimental conditions. In the water samples after the treatment, the contents of cyanobacteria were 710 cells/mL and 606 cells/mL for P_AC and A_AC, respectively. Those findings indicate that that water quality can be significantly improved, so that it can be classified as low risk according to WHO [77]. However, the results for C_AC show that the water still indicates a high level of risk, i.e., 422,534 cells/mL.

Namely, despite the higher S_BET_ value and the porous volume of C_AC, the reason for improved efficiency of prepared AC may be in the morphology of cyanobacteria and their compatibility with the distribution of micropores and mesopores in the structure of synthesized AC samples. Namely, in terms of morphology, cyanobacteria represent a diverse group, which includes unicellular forms, which can be very small, colonial forms and multicellular filamentous forms, with or without branching. Multicellular filamentous forms are dominant in Aleksandrovac Lake (e.g., *Rhapidiopsis raciborskii*, *Limnothrix planctonica, Jaaginema subtilissimum*). In filamentous forms, the cells remain adhered to each other after division, forming chains of connected cells termed “trichomes” (Table 6, Table 7 and Table 8). In some taxa, trichomes can be enveloped in a mucous sheath. The major components of this mucous surface structure in cyanobacteria are exopolysaccharides (glycans), comprising neutral sugars (such as glucose, galactose, and mannose), and uronic acids (mainly glucuronic and galacturonic), as well as their derivates containing amine, methyl, and sulfate groups (e.g., N acetylglucosamine) [78]. These components of mucous surface structures may be responsible for the binding process to the micro- and mesoporous structure of activated carbons and are one of the reasons for the high level of efficiency of synthesized ACs compared to microporous commercial activated carbon. Additionally, the cells within a single trichome of multicellular cyanobacteria can differ in size, form, or cell-wall composition. Each trichome can be composed of several tens, hundreds, or thousands of cells. The morphological differences between trichomes (number of constituent cells and presence/absence of rigid sheath and/or mucous layer) affect the process of removing the cells from the native sample. The length of the cyanobacterial trichomes plays an important role in the removal process, where longer trichomes are easier to retain than shorter trichomes.

The presented results indicate the extremely significant potential for the removal of cyanobacteria before any toxin production. In this way, it is possible to prevent any further contamination of the water with various potentially carcinogenic toxins, which can cause various diseases (Table 5). The applied activated carbons are characterized by a specific distribution and pore size (micropore and mesopore structure), which may be the reason for their high efficiency in removing cyanobacteria. However, the literature data have shown that activated carbons can be successfully applied to adsorb extracellular toxins [79], while the removal of cyanobacteria and intracellular toxins currently lacks detailed research. Namely, Silva Buarque et al. [80] studied the effect of pore size on the adsorption of saxitoxin, and it was found that mesopores played an essential role in promoting the adsorption capacity and adsorption kinetics of granular activated carbon from coconut. The granular activated carbon from coconut exhibited higher adsorption capacity and a higher pseudo-second-order rate constant, as it has an elevated amount of mesopores. Small pore diameters facilitated large surface areas [79], which improved the adsorption of contaminants.

In contrast, regarding STXs, other researchers have reported that microporous activated carbons have the greatest capacity to adsorb STXs [81]. However, most of the studies relating to the activated carbon adsorption of cyanotoxins have been conducted on the MCs, in particular MC-LR, which is considered the most common cyanotoxin [81]. Generally, activated carbon most effectively removes dissolved MCs from water, with yields of removal of up to 99%. Moreover, the MC variants may have different adsorption efficiencies; the order for the four variants from least to most adsorbent was reported to be MC-RR, MC-YR, MC-LR, and MC-LA [81].

Thus, the application of activated carbons can successfully adsorb extracellular toxins [81]; however detailed research on the manner and mechanisms of the removal of cyanobacteria and intracellular toxins by means of the application of active carbons is currently lacking.

By analyzing the trichal Cyanobacteria in the water from Aleksandrovac Lake before and after the treatment of activated carbons in August 2021 (Table 8) and comparing them with the Cyanobacteria dimensions from the literature (Table 7), it can be noted that the dimensions of the Cyanobacteria are significantly larger than the micropores and mesopores of activated carbons. Based on this, it can be concluded that the adsorption mechanism of Cyanobacteria cannot bind to that size of micropores or mesopores (Table 2). However, the literature data [68,81] have shown the possibility of increasing the densities of the functional groups near the surface of the activated carbon obtained from wood; it is clear that one of the methods of Cyanobacterial adsorption may be due to the interaction between the surface groups of applied materials and Cyanobacterial cells. The mentioned potential mechanism will be the subject of our innovative further research in this area and on the obtained materials.

The innovativeness of the obtained results also contributes to the fact that the literature data indicate that there are not enough data about using activated carbon materials in orders to remove cyanobacteria, as well as about the adsorption mechanism of cyanobacteria. Limited studies on the adsorption of cyanobacteria onto activated carbons [75,77,78,79,80,81] exist, which shows a clear need for more systematic studies to determine which type of activated carbon, dosage, pH, and contact time are appropriate. As a part of our next research, we have a plan to present the adsorption kinetics of A_AC and P_AC materials with different content of materials. The focus of our next research will be on achieving optimal cyanobacteria removal through the adsorption of A_AC and P_AC materials.

## 4. Conclusions

In order to overcome the problem of the presence of cyanobacteria in aquatic ecosystems, the synthesis of completely new activated carbons and their potential application in contaminated water treatment was successfully performed. The new active carbon materials obtained from waste biomass, date-palm leaf stalks (P_AC), and black alder cone-like flowers (A_AC) of reliable physical and chemical characteristics have been successfully synthesized. Their detailed characterization was carried out using XRD, FTIR, N_2_ physisorption, and FESEM methods. In order to complete the comparisons, the commercial activated carbon (C_AC) was also examined. The analysis of the adsorption properties of all activated carbon materials was conducted on water samples from the Aleksandrovac Lake (Southern part of Serbia), a eutrophic lake, in order to remove Cyanobacteria from water. The activated carbon materials showed removal efficiency of P_AC, A_AC, and C_AC of 99.99%, 99.99%, and 89.79%, respectively. Based on the obtained results, it can be concluded that this paper provides potential innovative solutions, from the possibility of utilizing waste biomass for the production of activated carbon materials to their application for the almost complete removal cyanobacteria through the adsorption from contaminated water before the release of cyanotoxins.

## Figures and Tables

**Table 1:** Lignocellulose composition of raw samples of precursor.

	Cellulose, %	Lignin, %	Hot Water Extractives, %	Hemicelluloses, %	Moisture, %	Ash (900 °C), %
**Date-palm Leaf Stalks**						
P_RS **	25.1 ± 0.7	7.9 ± 0.5	48.66 ± 0.04	1.31	7.9 ± 0.2 *	4.5 ± 0.3
Bendahou et al., 2007 [11]	44.0	14.0	/	28.0	/	2.5
Alotaibi et al., 2019 [12]	35.00	20.13	/	15.40	15.6	12.6
**Black Alder Cone-like Flowers**						
A_RS **	19.1 ± 1.2	29.6 ± 0.9	28.65 ± 1,14	10.67	10.5 ± 0.4 *	2.7 ± 0.1
Mokrzycki et al., 2019 [13]	20.9	29.7	/	29.3	6.4	2.6

* (wt.%, dry); ** with st. dev. (±).

**Table 2:** Textural characteristics of activated carbons using the various calculations methods.

		C_AC	P_AC	A_AC
Specific surface area (Brunauer–Emmett–Teller), Journal of the American Chemical Society. 60 (2), 309–319	C	1292	52	1287
	S, m^2^ g^−1^	1100	36.6	485
t-Plot (Lippens and de Boer); standard isotherms from literature: A. Lecloux, J.P. Pirard, J. Colloid Interface Sci. 70 (2), 1979	Total surface area, m^2^ g^−1^	1105	36.1	446
	Micropore volume, cm^3^ g^−1^	0.457	0.023	0.240
	Mesopores surface m^2^ g^−1^	6.3	3.4	10.4
Micropore volume (Dubinin and Raduskevich), cm^3^ g^−1^		0.422	0.012	0.186
Micropores (Horvath and Kawazoe), with potential function: N_2_ on Graphite @77.3 K from literature: G. Horvath, K. Kawazoe. J. Chem. Eng. Japan, 16, 6(1983), 470–475	Maximum pore diameter, nm	0.48	-	0.48
	Cumulative pore volume, cm^3^ g^−1^	0.431	-	0.195
Mesopore volume, cm^3^ g^−1^	Adsorption branch of isotherm	0.057	0.022	0.097
	Desorption branch of isotherm	0.099	0.029	0.125
Total pore volume (Gurvich), cm^3^ g^−1^	At p/p_0_ = 0.98	0.474	0.038	0.268

**Table 3:** Phytoplankton in Aleksandrovac Lake from December 2020 till November 2021.

**Cyanobacteria**
*Anabaenopsis elenkinii* V.Milleer
*Anathece minutissima* (West) Komárek, Kastovsky and Jezberová
*Aphanocapsa* Nägeli sp.
*Glaucospira* G.Lagerheim sp.
*Jaaginema subtilissimum* (Kütz. Ex De Toni) Anagn. et Kom.
*Limnothrix planctonica* (Wolos.) Meffert
*Merismopedia glauca* (Ehr.) Kütz.
*Microcystyis aeruginosa* Kütz.
*Microcystis flos-aquae* (Wittr.) Kirchner
*Oscillatoria limosa* C.Agardh ex Gomont
*Planktolyngbya limnetica* (Lemmermann) Komárková-Legnerová and Cronberg
*Pseudanabaena limnetica* (Lemm.) Komarek
*Raphidiopsis raciborskii* Woloszynska) Aguilera et al.
*Snowella* Elenkin sp.
*Synechocystis* cf. *aquatilis* Sauvageau
**Dinophyta**
*Gymnodinium* Stein sp.
*Peridiniopsis* Lemm. sp.
*Peridiniopsis cunningtonii* Lemm.
**Chrysophyta**
*Mallomonas* Perty sp.
*Ochromonas* Vysotskii (Wysotzki, Wyssotzki) sp.
**Cryptophyta**
*Cryptomonas* Ehr. spp.
*Rhodomonas lacustris* Pascher and Ruttner
**Bacillariophyta**
*Achnanthidium* Kützing sp. (cf. *minutissimum*)
*Amphora lybica* Ehrenberg
*Cyclotella* (Kütz.) Bréb.sp.
*Cymbella* C.Agardh. sp.
*Fragilaria ulna* Sippen *acus* sensu L.-B.
*Gomphonema* Ehr. sp.
*Navicula* Bory sp.
*Stephanodiscus* Ehr. sp.
**Chlorophyta**
*Chlamydomonas* Ehr. sp.
*Chlorella* Beij. sp.
*Cosmarium* Corda sp.
*Kirchneriella contorta* (Schmidle) Bohl.
*Kirchneriella irregularis* (G.M.Smith) Korshikov
*Monoraphidium contortum* (Thur.) Com.-Legn.
*Mougeotia* C.Agardh sp.
*Scenedesmus quadricauda* (Turp.) Bréb.
*Scenedesmus semprevirens* Chod.
*Sphaerocystis* Chodat sp.
*Staurastrum* Meyen ex Ralfs sp.
*Tetraedron minimum* (A.Br.) Hansg
**Euglenophyta**
*Euglena* Ehr. sp.
*Euglena acus* Ehr.
*Phacus pyrum* (Ehr) W.Archer
*Trachelomonas volvocina* Ehr.

**Table 4:** Cyanobacteria detected in Aleksandrovac Lake in 2020 and 2021 and potential present cyanotoxins.

No	Cyanobacteria	Cyanotoxins	Reference
1.	*Anabaenopsis elenkinii*	*not determined*	*/*
2.	*Anathece minutissima*	*not determined*	*/*
3.	*Aphanocapsa* sp.	microcystin	[70]
4.	*Glaucospira* sp.	*not determined*	*/*
5.	*Jaaginema subtilissimum*	*not determined*	*/*
6.	*Limnothrix planctonica*	*not determined*	*/*
7.	*Merismopedia glauca*	*not determined*	*/*
8.	*Microcystyis aeruginosa*	anatoxin, microcystin	[70]
9.	*Microcystis flos-aquae*	microcystin	[70]
10.	*Oscillatoria limosa*	microcystin	[70]
11.	*Planktolyngbya limnetica*	*not determined*	*/*
12.	*Pseudanabaena limnetica*	Anatoxin	[70]
13.	*Rhapidiopsis raciborskii*	cylindrospermopsin, saxitoxin	[70]
14.	*Snowella* sp.	*not determined*	*/*
15.	*Synechocystis* cf. *aquatilis*	*not determined*	*/*

**Table 5:** Cyanotoxins, their chemical structures, molecular weights, effects, and producers.

Cyanotoxin	Chemical Structure	Molecular Weight	Effect
Anatoxin	Bicyclic alkaloid	252 Da [70], 165 and 179 Da [71]	Neurotoxin, inhibits acetylcholine esterase [72,73]
Cylindrospermopsin	Tricyclic guanidine alkaloid	415 Da [72]	Toxic effect on multiple organs; neurotoxic, genotoxic, protein synthesis inhibitor, hepatotoxin [72,73]
Microcystin	Cyclic peptides	from 800 to 1100 Da [72]	Hepatotoxic, tumor-causing, inhibition of eukaryotic phosphatase proteins PP1, PP2A, as well as phosphoprotein phosphatase PPP4, PPP5 [72,73]
Saxitoxin	Alkaloids	from 241 to 491 [72]	Neurotoxic, blocks sodium transport channels [72,73]

**Table 6:** Cyanobacteria analyses from Aleksandrovac Lake in August 2021 and removal efficiency of activated carbons.

No.	Cyanobacteria	Before Treatment	After Treatment		
			P_AC	A_AC	C_AC
		[cell/mL]	[cell/mL]		
1.	*Anabaenopsis elenkinii*	280	0	0	0
2.	*Anathece minutissima*	1280	0	0	0
3.	*Aphanocapsa* sp.	640	0	0	247
4.	*Glaucospira* sp.	49,150	143	93	20,876
5.	*Jaaginema subtilissimum*	1,050,812	0	193	15,825
6.	*Limnothrix planctonica*	1,018,116	463	194	6206
7.	*Merismopedia glauca*	842,052	0	0	362,880
8.	*Microcystyis aeruginosa*	63,193	0	0	0
9.	*Microcystis flos-aquae*	17,554	0	0	0
10.	*Oscillatoria limosa*	14,043	0	0	0
11.	*Planktolyngbya limnetica*	129,898	0	0	0
12.	*Pseudanabaena limnetica*	59,683	0	28	10,584
13.	*Rhapidiopsis raciborskii*	1,330,286	104	98	5916
14.	*Snowella* sp.	484,483	0	0	0
15.	*Synechocystis* cf. *aquatilis*	31,597	0	0	0
	***SUM***	**5,093,067**	**710**	**606**	**422,534**
	**The removal efficiency (%)**		**99.99**	**99.99**	**89.79**

**Table 7:** Dimensions of cyanobacterial cells from literature data.

No.	Cyanobacteria	Cell Shape	Length (µm)	Diameter/Width (µm)	Talus Type	Trichome Length (µm)	References
**1.**	***Anabaenopsis elenkinii***	ellipsoid	5.5–9.3 (3) 4–9 (12.9)	(2.8) 4–6(8)	trichome	≤100	[34,79,80,81]
**2.**	***Anathece minutissima***	ellipsoid	1.5 1–1.5–2 (?2.8)	0.9 0.8–1 (1.6)	colony	/	[79,80] [32]
**3.**	***Aphanocapsa* sp.**	sphere	/	1–1.5 *	colony	/	[32,82]
**4.**	***Glaucospira* sp.**	cylindrical	0.5–2	2.7–6	trichome	36–60	[83]
**5.**	***Jaaginema subtilissimum***	cylindrical	Up to 5 µm	1–1.5	trichome	150–300	[33,84]
**6.**	***Limnothrix planctonica***	cylindrical	2–4 6–10	1.5–2.5 (3)	trichome	/	[33,79,80,85]
**7.**	***Merismopedia glauca***	sphere	/	(2.8) 3–6	colony	/	[32,79,80]
**8.**	***Microcystyis aeruginosa***	sphere	/	4–6	colony	/	[32,79,80]
**9.**	***Microcystis flos-aquae***	sphere	/	(2.5)3.5–4.8 (5.6?)	colony	/	[32,79,80]
**10.**	***Oscillatoria limosa***	cylindrical	1.8–3 1.5–5(6)	10 (6?–9) 10–20 (22)	trichome	10–100	[86,87] [33]
**11.**	***Planktolyngbya limnetica***	cylindrical	1–2–5 (9?)	0.5–1–1.8 (2.5?)	trichome	/	[33,79,80]
**12.**	***Pseudanabaena limnetica***	cylindrical	5-14 (1.2?) 4–12 (20?)	(1) 1.2–1.5 (2)	trichome	/	[33,79,80,88]
**13.**	***Rhapidiopsis raciborskii***	cylindrical	2.3–6.5 2.5–12 (16)	(1.7) 2–2.4 (−4?)	trichome	107, 50–300	[19,89,90,91]
**14.**	***Snowella* sp.**	sphere or elipsoid	2.4–4	3.2	colony	/	[32,79,80]
**15.**	***Synechocystis aquatilis***	sphere	/	(3) 4.5–7 (7.8?)	colony	/	[32,92]

* The diameter of the *Aphanocapsa* species may be larger, but the values of the species in Aleksandrovac Lake were generally up to 1.5 µm in diameter.

**Table 8:** Analysis of trichomal Cyanobacteria in water from Aleksandrovac Lake before and after treatment of activated carbons in August 2021.

No.	Cyanobacteria	Cell One Length	Average Trichomes Length before Treatment	Average Trichomes Length after Treatment		
				P_AC	A_AC	C_AC
**1.**	***Anabaenopsis elenkinii***	7 µm	30 µm	/	/	/
**2.**	***Glaucospira* sp.**	1.5 µm	35 µm	23–30 µm	21–35 µm	21–36 µm
**3.**	***Jaaginema subtilissimum***	1.7 µm	48 µm	20–41 µm	18–45 µm	21–48 µm
**4.**	***Limnothrix planctonica***	5 µm	59 µm	16–21 µm	13–27 µm	15–29 µm
**5.**	***Oscillatorialimosa***	3 µm	37 µm	30–35 µm	/	32–35 µm
**6.**	***Planktolyngbya limnetica***	2 µm	17 µm	/	/	/
**7.**	***Pseudanabaena limnetica***	5 µm	141 µm	38–63 µm	32–59 µm	39–62 µm
**8.**	***Rhapidiopsis raciborskii***	7 µm	71 µm	27–41 µm	15–45 µm	25–47 µm

## Data Availability

Not applicable.

## Supplementary Materials

The following supporting information can be downloaded at: https://www.mdpi.com/article/10.3390/ijerph19116639/s1, File S1: A detailed description of the eutrophication stages that take place in the lake. File S3-Figure S1: The original pages of the report that contain the distribution of micropores according to the Horvat-Kavoazo model; File S2-Table S1: The ratios of the specific volume of micropores (V_mic_) were calculated by different methods and the specific volumes of mesopores calculated from the adsorption V_meso_ (ads) and desorption Vmeso (des) branches; File S4-Table S2: Quantitative analysis of Cyanobacteria in Aleksandrovac Lake during 2020.; File S5-Table S3: Quantitative analysis of Cyanobacteria in Aleksandrovac Lake during 2021.
