# Supplementary material for: Natural Cyanobacteria Removers Obtained from Bio-Waste Date-Palm Leaf Stalks and Black Alder Cone-Like Flowers
Source: Int J Environ Res Public Health. 2022 May 29;19(11):6639. doi: 10.3390/ijerph19116639 (PMC9180351; doi:10.3390/ijerph19116639)
Supplement: Supplementary file 1 [file ijerph-19-06639-s001.zip › Supplementary materilas_S1.pdf]

S1_A detailed description of the eutrophication stages that take place in the lake.

1. Introduction

The Aleksandrovac Lake is an artificial accumulation (area about 120 000 m^2, built for irrigation purposes on the river Aleksandrovac in 1964), which is supplemented with water from the local Otulja river. The eutrophication processes has been significantly increased during the time leading to highly eutrophic and almost anaerobic conditions. Over the time, due to increased activity and inflow of nutrients in the basin there was an intensive eutrophication of the lake. This is primarily due to inflow of nutrients from the basin, the relatively small depth of the lake (in some places under 2 m), due to drying up of the local Otulja River, as well as due to natural factors and climatic characteristics. These parameters were suitable for cyanobacteria bloom over the time. Eutrophication and climate change are two the most common factors that can affect the increase in volume, population density and persistence of cyanobacteria blooms which could result in fish mortality [14].

There were two massive fishing mortalities, in 2008 with a consequent loss of over 3 000 kg of the fish. The possible reason was change of pH of lake water and lowering oxygen concentration, as well as toxin production by cyanobacteria [15]. Rapid eutrophication, forced efforts for improving the water quality, and according to the Main Project "Rehabilitation of the Aleksandrovac Lake" (July 2009) proposed by the Institute for Water Management "Jaroslav Cherni" - Belgrade (Department of Dams, hydropower, mines and roads), the lake was emptied by the end of 2009, and the reconstruction program began in the spring of the 2010 [16]. The lake was refilled with water, and then used for tourism and recreation during the summer of 2010. Results of phytoplankton diversity of lake was monitored during 2008 (before rehabilitation), as well as 2011 (after rehabilitation), and were presented in paper Đorđević and Simić, 2014 [15], where the presence of Cyanobacteria was confirmed. Further, a new massive fishing mortality in Aleksandrovac Lake was in mid-December 2012. Local residents reported thousands of dead fish floating on the water. Almost entire fish population was lost (more than 1.7 tonnes), including carp (*Cyprinus carpio*), wells catfish (*Silurus glanis*), grasscarp (*Ctenopharyngodon idella*), silver carp (*Hypophthalmichthys molitrix*), common bream (*Abramis brama*), gibel carp (*Carassius gibelio*), asp (*Aspius aspius*) and chub (*Squalius cephalus*). Additionally, several weeks before the incident, a cyanobacterial species *Cylindrospermopsis raciborskii* was noted, which gave a yellow/brown colour of the water. The tropical invasive potential toxic cyanobacteria species *Raphidiopsis raciborskii* (Woloszynska) Aguilera & al. 2018 (formerly *Cylindrospermopsis raciborskii* (Woloszynska) Seenayya & Subba Raju 1972) is known as spreading areal into moderate climate region. *R. raciborskii* produces several toxins: cilindrospermopsin, saxitoxins and anatoxin [17]. The way these species can survive in moderate climate is by ackinetes (dormant cells of cyanobacteria species) and it helps to tolerate unfavourable conditions and the main cause of their invasion [18]. The presence of *R. raciborskii* can cause degradation of biodiversity and displacement of native phytoplankton species as an invasive species. For the first time, this species was recorded in Serbia and was described in 2006 by Cvijan and Fužinato et al., 2011 in Slatina Pond [19], and the first blooming was recorded in Ponjavica river in 2008 [20]. Control of the presence and abundance of this species in many countries is extremely important for water quality management since the proliferation of this species never forms a surface bloom, so it cannot be detected by visual monitoring. The *R. raciborskii* shows wide tolerance to temperature values; it is capable to sustain biomass at temperatures as low as 11-17 °C, and up to 35 °C which was the highest temperature that was observed when the population increase [21].

Detailed results of the various samples (water, sediment and fish tissue) taken from Aleksandrovac Lake in the mentioned period are given in paper Svirčev et al., 2016 [14]. From the aspect of analysis of the presence of cyanobacteria, 15 November 2012, counting of filaments was performed only on dominating species *R. raciborskii* and the average value per sample was 2.06×10^5 filaments per mL [14]. In December 2012 *R. raciborskii* was also dominant and the number of filaments per mL was 1.24×10^5 [14]. Due to presented results this lake was chosen as an example of lake suitable for testing the possibility of application of new activated carbon materials.
