# Supplementary material for: Natural Cyanobacteria Removers Obtained from Bio-Waste Date-Palm Leaf Stalks and Black Alder Cone-Like Flowers
Source: Int J Environ Res Public Health. 2022 May 29;19(11):6639. doi: 10.3390/ijerph19116639 (PMC9180351; doi:10.3390/ijerph19116639)
Supplement: Supplementary file 1 [file ijerph-19-06639-s001.zip › Supplementary materilas_S2.pdf]

S1_Additional information about textural properties.

Table S1. The ratios of the specific volume of micropores (V_{mic}) were calculated by different methods and the specific volumes of mesopores calculated from the adsorption V_{meso} (ads) and desorption Vmeso (des) branches.

	C_AC			A_AC		
**V_{mic} metod calculation**	t-plot	DR [27]	HK [28]	t-plot	DR [27]	HK [28]
**V_{mic}/V_{meso}(ads)**	8.0	7.4	7.6	2.5	1.9	2.0
**V_{mic}/V_{meso}(des)**	4.6	4.3	4.4	1.9	1.5	1.6
