# Supplementary material for: Natural Cyanobacteria Removers Obtained from Bio-Waste Date-Palm Leaf Stalks and Black Alder Cone-Like Flowers
Source: Int J Environ Res Public Health. 2022 May 29;19(11):6639. doi: 10.3390/ijerph19116639 (PMC9180351; doi:10.3390/ijerph19116639)
Supplement: Supplementary file 1 [file ijerph-19-06639-s001.zip › Supplementary materilas_S3.pdf]

Micropores (Horvath and Kawazoe)

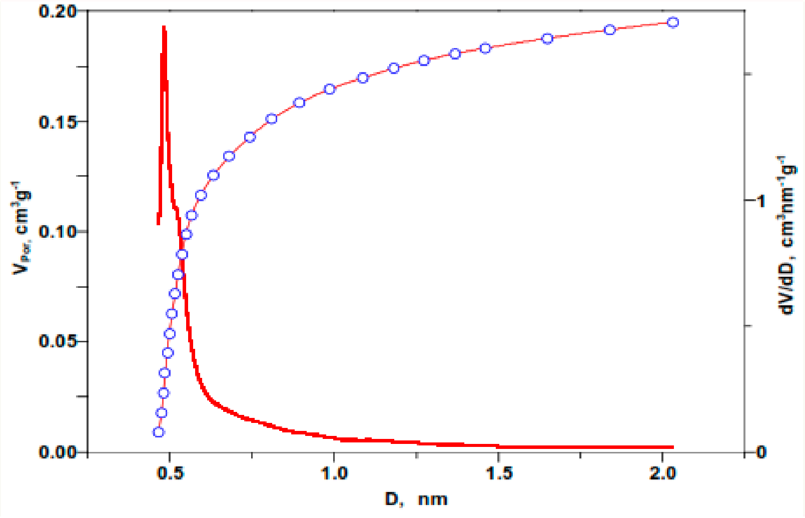

2021.03.24. A_AC BI 1-0.38

Calculations from p/p^0 0 to 0.2

with potential function: Nitrogen on Graphite @77.3 K

from literature: G. Horvath, K. Kawazoe, J. Chem. Eng. Japan, 16, 6(1983), 470-475

Calculation with a molecular area of	16.2 $Å^2$
molecular weight of	28.01 g/mol
and liquid density of	0.8086 g cm^{-3}
Median pore diameter	0.5473 nm
Maximum pore diameter	0.4825 nm
Cumulative pore volume	0.1949 cm^3g^{-1}

Micropores (Horvath and Kawazoe)

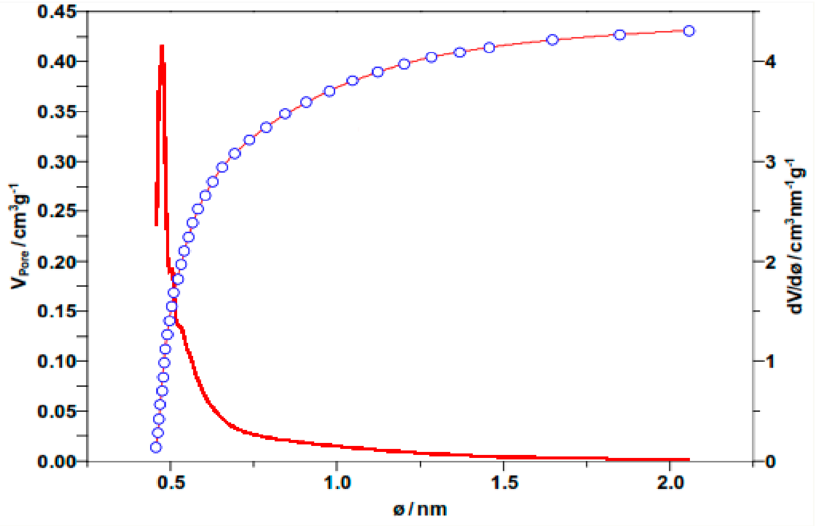

2021.03.12. C_AC BI 1-0.38

Calculations from p/p^0 0 to 0.2

with potential function: Nitrogen on Graphite @77.3 K

from literature: G. Horvath, K. Kawazoe, J. Chem. Eng. Japan, 16, 6(1983), 470-475

Calculation with a molecular area of	16.2 $Å^2$
molecular weight of	28.01 g/mol
and liquid density of	0.8086 g cm^{-3}
Median pore diameter	0.5461 nm
Maximum pore diameter	0.4753 nm
Cumulative pore volume	0.4309 cm^3g^{-1}

Figure S1. The original pages of the report that contain the distribution of micropores according to the Horvat-Kavoazo model.
