# Supplementary material for: Natural Cyanobacteria Removers Obtained from Bio-Waste Date-Palm Leaf Stalks and Black Alder Cone-Like Flowers
Source: Int J Environ Res Public Health. 2022 May 29;19(11):6639. doi: 10.3390/ijerph19116639 (PMC9180351; doi:10.3390/ijerph19116639)
Supplement: Supplementary file 1 [file ijerph-19-06639-s001.zip › Supplementary materilas_S4.pdf]

S2_Quantitative analysis of Cyanobacteria in Aleksandrovac Lake during 2020.

Table S2. Quantitative analysis of Cyanobacteria in Aleksandrovac Lake during 2020.

**Year**	**2020**											
**Month**	**January**	**February**	**March**	**April**	**May**	**June**	**July**	**August**	**September**	**October**	**November**	**December**
**Cyanobacteria**	[cell/mL]											
Anabaenopsis elenkinii	1652	0	0	0	5507	5120	4800	5199	2599	0	2599	4130
Anathece minutissima	0	0	11328	14160	18880	23405	10971	17824	11883	0	0	0
Aphanocapsa sp.	11328	1634	7552	9440	6293	35107	10971	11883	17824	11883	5941	37760
Glaucospira sp.	16363	8169	141757	145927	70144	210645	50286	1222785	1071655	200517	26983	52117
Jaaginema subtilissimum	110504	20824	86487	77221	494906	377534	320108	275439	317814	40628	155521	121471
Limnothrix planctonica	208860	331943	215232	242136	208860	517542	888686	353356	380538	256291	418487	137883
Merismopedia glauca	1416	5991	1416	1770	4720	8777	4114	5941	14853	11883	2228	3540
Microcystyis aeruginosa	0	0	0	0	0	5851	10971	2971	0	0	5941	0
Microcystis flos-aquae	0	0	0	0	0	5851	5486	2971	5941	0	0	0
Oscillatoria limosa	7080	0	18880	23600	41300	25599	24686	14482	14482	27478	26736	0
Planktolyngbya limnetica	7080	0	28084	25075	82600	0	10286	47530	11883	5941	0	8260
Pseudanabaena limnetica	79296	85777	10384	6490	147107	40959	10286	9655	4827	5199	100259	429520
Raphidiopsis raciborskii	37018	2781	96423	106832	87882	666101	1425306	1882212	1186793	467874	106200	37974
Snowella sp	2360	4357	2360	1475	7867	11702	10971	11883	17824	22280	11883	9440
Synechocystis	1652	681	4956	2065	11013	0	9600	2228	4456	0	0	4720
SUM	**484608**	**462156**	**624859**	**656190**	**1156399**	**1934194**	**2797528**	**3866357**	**3063370**	**1049973**	**862779**	**846814**
