# Supplementary material for: Natural Cyanobacteria Removers Obtained from Bio-Waste Date-Palm Leaf Stalks and Black Alder Cone-Like Flowers
Source: Int J Environ Res Public Health. 2022 May 29;19(11):6639. doi: 10.3390/ijerph19116639 (PMC9180351; doi:10.3390/ijerph19116639)
Supplement: Supplementary file 1 [file ijerph-19-06639-s001.zip › Supplementary materilas_S5.pdf]

S3_Quantitative analysis of Cyanobacteria in Aleksandrovac Lake during 2021.

Table S3. Quantitative analysis of Cyanobacteria in Aleksandrovac Lake during 2021.

**Year**	**2021**											
**Month**	**January**	**February**	**March**	**April**	**May**	**June**	**July**	**August**	**September**	**October**	**November**	**December**
**Cyanobacteria**	[cell/mL]											
Anabaenopsis elenkinii	2065	3304	4956	0	8260	6144	4800	280	5199	2599	2599	0
Anathece minutissima	0	0	0	9440	28320	28086	32914	1280	17824	0	0	4720
Aphanocapsa sp.	47200	15104	11328	9440	9440	42129	10971	640	23765	5941	11883	9440
Glaucospira sp.	62540	24544	40907	177197	105217	252774	125714	49150	1249256	401035	40475	140420
Jaaginema subtilissimum	173529	138129	207194	108109	742359	453041	280094	1050812	360189	60942	194401	104429
Limnothrix planctonica	156026	231138	208860	269040	313290	621051	938057	1018116	394128	300479	461190	225814
Merismopedia glauca	1770	2832	2832	1770	7080	10532	8229	842052	17824	11883	4456	1770
Microcystyis aeruginosa	0	0	0	0	0	7021	5486	63193	0	0	2971	2950
Microcystis flos-aquae	0	0	0	0	0	7021	5486	17554	8912	0	0	0
Oscillatoria limosa	0	7080	14160	23600	61950	30719	49371	14043	14482	13739	13368	20650
Planktolyngbya limnetica	4130	7080	7080	35105	123900	0	30857	129898	17824	11883	0	14160
Pseudanabaena limnetica	414180	92512	49560	12980	220660	49150	20571	59683	19309	10397	94689	61950
Raphidiopsis raciborskii	44303	41131	71980	120529	131823	799321	1316529	1330286	1222785	623832	159300	132160
Snowella sp	4720	3540	4720	2950	5900	14043	10971	484483	29706	89119	23765	14160
Synechocystis	0	3304	1652	6195	8260	0	4800	31597	2228	0	3713	4425
SUM	**910463**	**569699**	**625229**	**776354**	**1766458**	**2321032**	**2844850**	**5093067**	**3383430**	**1531849**	**1012810**	**737048**
